# Microbiological Monitoring of the Environment Using the “Association Rules” Approach and Disinfection Procedure Evaluation in a Hospital Center in Morocco

**DOI:** 10.1155/2021/7682042

**Published:** 2021-07-05

**Authors:** Rachid Flouchi, Abderrahim Elmniai, Mohamed El Far, Ibrahim Touzani, Naoufal El Hachlafi, Kawtar Fikri-Benbrahim

**Affiliations:** ^1^Laboratory of Microbial Biotechnology and Bioactive Molecules, Science and Technologies Faculty, Sidi Mohamed Ben Abdellah University, Fez, Morocco; ^2^High Institute of Nursing Professions and Health Techniques Annex Taza, Fez, Morocco; ^3^Human Pathology, Biomedicine and Environment Laboratory, Faculty of Medicine and Pharmacy, Sidi Mohamed Ben Abdellah University, Fez, Morocco; ^4^Provincial Laboratory of Epidemiology and Environmental Hygiene, DMS Taza, Taza, Morocco; ^5^Laboratory of Applied Physics, Computer Science and Statistics Sciences Faculty Dhar El Mahraz, Sidi Mohamed Ben Abdellah University, Fez, Morocco

## Abstract

**Background:**

The hospital environment, especially surfaces and medical devices, is a source of contamination for patients.

**Objective:**

This study carried out, to the best of our knowledge, for the first time at Taza Hospital in Morocco aimed to assess the microbiological quality of surfaces and medical devices in surgical departments and to evaluate the disinfection procedure in time and space.

**Methods:**

Samples were taken by swabbing after cleaning the hospital surface or medical device, to isolate and identify germs which were inoculated on semiselective culture media then identified by standard biochemical and physiological tests, using the analytical profile index (API) galleries. Moreover, the association rules extraction model between sites on the one hand and germs on the other hand was used for sampling.

**Results:**

The study showed that 83% of the samples have been contaminated after biocleaning. The most contaminated services have been men's and women's surgeries. 62% of isolated germs have been identified as Gram-positive bacteria, 29% as Gram-negative bacteria, and 9% as fungi. Concerning the association rules extraction model, a strong association between some contaminated sites and the presence of germ has been found, such as the association between wall and nightstand and door cuff, meaning that the wall and nightstand contamination is systematically linked to that of the door cuff. The disinfection procedure efficacy evaluation has enabled suggesting renewing it each 4 h.

**Conclusion:**

Microbiological monitoring of surfaces is necessary at hospital level through the use of the association rule extraction model, which is very important to optimize the sampling, cleaning, and disinfection site scenarios of the most contaminated ones.

## 1. Introduction

The main prerogative of hospital institutions is to provide care for patients. Unfortunately, some complications can emerge in certain cases as nosocomial infections (NAI), which include any infectious event related to the individuals' care. These infections affect approximately 5% to 10% of patients in developed countries but the risk is 2 to 20 times higher in developing countries [1]. The presence and increase of these infections induce an extension in the length of stay of patients in hospital, overuse of antibiotics leading sometimes to the emergence of resistant microorganisms, increased costs and expenses, either for the patient or the hospital, and a high mortality rate [2].

Among the main causes of these infections, the patient's environment, particularly the hospital rooms' surfaces and architecture, as well as the medical or accommodation equipments frequently used, presents a risk of germ's transmission and contamination by germs in the hospital environment [3]. Indeed, surfaces in the immediate vicinity of patients are frequently contaminated by the hands of hospital patients and health care professionals and are classified as high-contact surfaces [4]. The most simple example concerns pill boxes which have been reported to contain *Staphylococcus* species (*S. captis, S. epidermis, S. hominis*…), *Corynebacterium striatum*, *Streprococcus sanguinis,* and *S. oralis*, representing thereby a source of high cross-contamination in hospitals and a risk factor for patients [5]. This risk is linked to the ability of coagulase-negative *Staphylococcus* species to produce a slimy biofilm enabling their adhesion to medical devices such as prosthetic valves and catheters which makes them the most common cause of prosthetic valve endocarditis, on the one hand [6]. *Corynebacterium striatum* gaining resistances to many antibiotics [7] has recently been recognized as an emerging pathogen, particularly in a nosocomial setting [8] where it could result in sepsis, on the other hand. Finally, *S. sanguinis* is also a common cause of subacute bacterial endocarditis; and *S. oralis* are opportunistic pathogens.

Furthermore, the ability of microorganisms to persist on environmental surfaces in hospitals varies greatly depending on the individual's biological factors, and the presence of organic matter or moisture on surfaces; moreover, these microorganisms causing nosocomial infections can remain alive for several weeks to months on dry surfaces [9]. Thus, several studies have shown that large numbers of germs have been isolated from door cuff and hospital floors [10]. Indeed, several types of germs of nosocomial origin have been isolated from surfaces and have shown an innate ability to survive on high-contact surfaces, such as methicillin-resistant *Staphylococcus aureus* [11], *Klebsiella pneumoniae* [12], coagulase-negative *Staphylococci* [13], and *Acinetobacter baumannii* [14].

Among the most well-known nosocomial infections, surgical site infections (SSI) are ranked second to urinary tract infections [15]. These SSIs develop from environment in the operating rooms during surgery through direct and multiple contacts between patients. The level of surface contamination in hospital wards differs according to the surface's and/or room's types, or the nature of hygiene in a specific unit [16].

Therefore, Centers for Disease Control and Prevention (CDC) have recommended the implementation of procedures, through the use of various monitoring tools that ensure effective cleaning and consistent disinfection of surfaces located close to the patient or likely touched by the patient and the health care professionals [17]. However, there are few studies in the literature on the actual assessment of environmental cleanliness in Moroccan hospitals. In this context, the purpose of this study is to identify and evaluate the pathogenic bacterial contamination of surfaces, medical equipment, and technical medical equipment in surgical departments in a hospital center in Taza (Morocco), to look for the association rules between germs and sampling points, and finally to evaluate the disinfection procedure using three selected disinfectants.

## 2. Materials and Methods

### 2.1. Study Site

Our study was carried out, during 12 months from June 2018 to May 2019, in the specialized surgery departments, at the provincial hospital of Taza (Fez-Meknes region, North Eastern Morocco) having a bedding capacity of 317 beds.

### 2.2. Sampling Point and Method

Microbiological sampling of surfaces was carried out according to ISO 14698-1 [18] and concerned the surfaces of patient wards and operating theaters. It was carried out outside of human activities and in postcleaning in the hospital ward and between surgical operations in the operating theaters. For this purpose, a surface area of 25 cm^2^ has been rubbed with a sterile cotton swab moistened by Brain Heart Infusion BHI, using the method of repeated striations in two zigzag directions. In order to obtain representative results, the sampling from each site was repeated three times [19].

The selected points were those closest to the patients and most frequently used in the hospital ward (bed rails, nightstand, ground, walls, and door cuff), and in the operating theater (operating table, wall, floor, operating theater, electric scalpel, and trolleys). The samples taken, 85 in total, were rapidly transported in a cooler, kept at 4°C, to the public health laboratory of the delegation Taza health ministry.

### 2.3. Cultivation, Isolation, and Identification of Germs

Culture and isolation have been performed by inoculating semiselective culture media: Mac Conkey (for Gram-negative bacilli and *Enterobacteriaceae*), Chapman (for *Staphylococci* and *Micrococci*), Cetrimide Agar (*Pseudomonas aeruginosa*, *Klebsiella*, and *Acinetobacter*), EMB for *Escherichia coli*, Blood Agar and Slanetz Bartley for Streptococci, and Sabouraud added with chloramphenicol for fungi (moulds and yeasts).

Inoculated Petri dishes were incubated at 30–37°C for 24 to 48 hours for bacterial cultures, and at 28–32°C, 5 to 7 days for fungi. The identification of bacteria was carried out according to the classical bacteriological methods, while macroscopic and microscopic examinations were carried out for moulds.

Hence, bacterial identification was based on morphological and biochemical characteristics. The preliminary identification tests concern colony appearance and microscopic examination after Gram staining for all isolated strains, and then lactose fermentation and oxydase activity were tested for bacilli selected on Mac Conkey agar; mannitol fermentation, catalase activity, coagulase, and DNase tests were performed for cocci isolated on chapman medium; a catalase test and a verification on Bile Esculin Agar (BEA) were performed for bacteria of the genus *Streptoccus*, while lactose and mannitol fermentations, catalase, and oxydase activities and motility were tested for bacteria isolated on cetrimide.

Thereafter, identification was continued by standard biochemical and physiological tests, carried out by the analytical profile index (API) galleries (API 20E® and API 20 NE), based on standardized, miniaturized biochemical tests enabling easy and fast identification of relevant bacteria. These tests are carried out in wells containing dehydrated substrates to detect different enzymatic activities of the tested microorganisms, related to the fermentation of some selected sugars, and to the catabolism of certain substances such as proteins, amino acids, or carboxylic acids. Briefly, bacterial suspensions with a suitable density are distributed in each well of the microgallery and the system is incubated at a suitable temperature (generally 30–37°C for 24–48 h). The metabolites produced during the incubation period are brought out by spontaneous color changes or revealed by the addition of specific reagents. The obtained results are compiled according to a standard table of reactions to obtain a strain's profile number. Finally, analytical catalogs are used to identify the bacterial species, while ensuring the identification authenticity.

Concerning fungal identification, a macroscopic study of colony characteristics enabled differentiating between fungi and yeasts grown on Sabouraud + chloramphenicol medium; then the filamentation test was performed by microscopic examination to confirm the presence of *Candida* for colonies having yeasts characteristics.

All identified strains were kept in glycerol tubes at −18°C.

### 2.4. Extraction Model Approach of the Association Rules

It is a model based on a transactional database, where items are represented in columns and transactions in lines. To adopt this model in our case study, we went through the realization of a hollow matrix where rows represent the isolated germs and columns represent the sampling points; this matrix serves as a source of the transactional database.

The technique adopted in this study consists of two steps:

- First, the algorithm a priori, which is part of the DATA maining (associative) algorithm, has been applied [20]. It remains always the most popular used algorithm thanks to its performance and efficiency. The algorithm performance parameters in our case: min support 25%, min confidence 75%.
- Second, our algorithm extracts association rules from different points of the chosen samples and the isolated germs according to the previous indicated parameter min of confidence index. To have important association rules, the left indicator which is strictly superior to 1 was applied.

### 2.5. Evaluation of the Effectiveness of the Disinfection Procedure in Time and Space

To evaluate the disinfection procedure, the most contaminated points have been chosen (ground surface), and 3 samples were taken (ground surfaces 1, 2, and 3) before and after disinfection by the studied disinfectants. The samples have been repeated each two hours to perform germs identification.

Three selected disinfectants belonging to the quaternary ammonium family were used to identify and evaluate their antibacterial activity.

## 3. Results

Based on 85 samples, 70 have been used to identify germs in surfaces and medical devices and 15 to assess the inhibitory potential of disinfectants against microorganisms. For the 70 surveillance samples taken from different points close to the patient, either in the hospital ward or operating theater, a contamination percentage of 83% (*n* = 58) has been found, compared to 17% (*n* = 12) uncontaminated samples.

**Figure 1:**
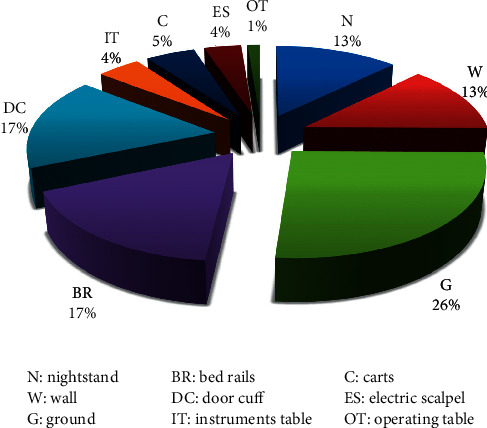
Contamination rate of the studied sampling sites.

The most contaminated sampling sites have been, respectively, the floors (26%), bed barriers and door handles (17%), and walls and bedside tables (13%), while the other sites represent less than 5% (Figure 1).

### 3.1. Distribution of Germs according to Sampling Points

**Figure 2:**
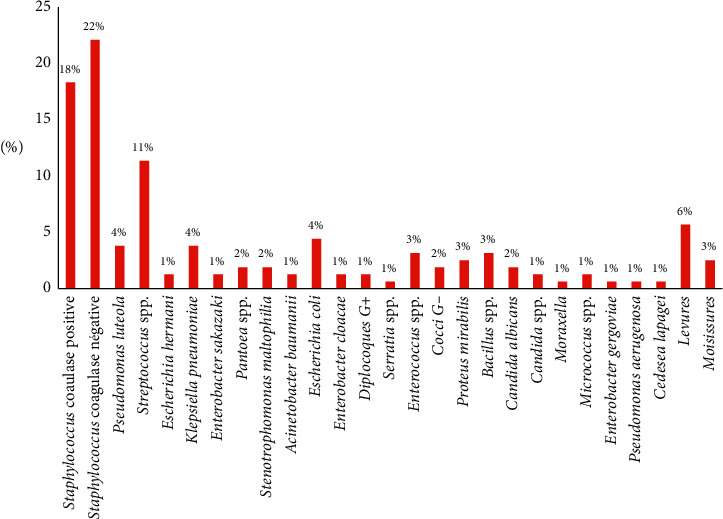
Distribution of isolated germs in the sampling sites.

**Figure 3:**
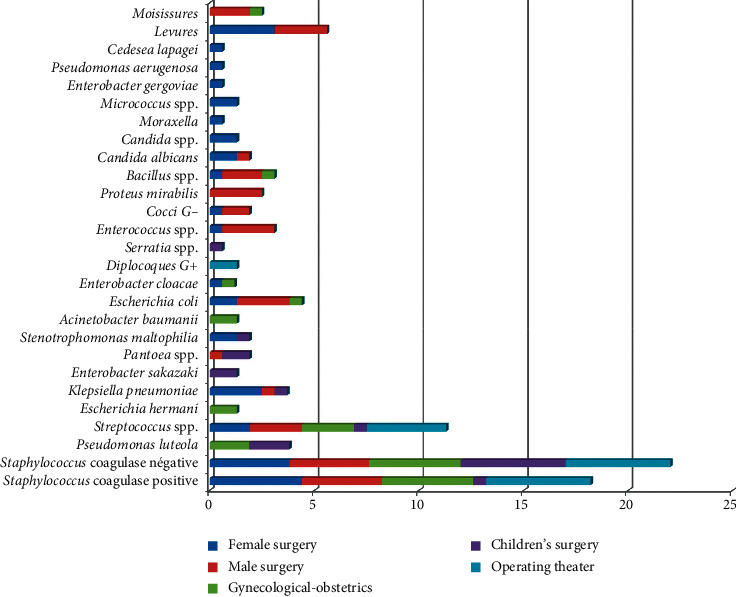
Distribution of isolated germs in the sampling sites according to each department.

A total of 27 microorganisms have been isolated from different sampling points consisting of 9 Gram-positive bacteria, 16 Gram-negative bacteria, and 2 fungi (yeasts and moulds). Concerning these germs' distribution, a predominance of Gram-positive bacteria has been noticed by 62%, followed by Gram-negative bacteria (29%) and then fungi (9%). The majority of these species belong to Staphylococcus species (40%), distributed between coagulase-negative *Staphylococci* (22%) and *Staphylococcus aureus* (18%), followed by Streptococci species (11%), *Pseudomonas luteola*, *Klebsiella pneumoniae*, and *Escherichia coli* (4%), and then *Enterococcus* spp., *Bacillus* spp., *Proteus mirabilis* (3%), *Pantoea* spp., *Stenotrophomonas maltophilia*, *Candida albicans*, and Gram-negative cocci (2%). The other bacteria represent less than or almost 1%, while the 9% of the fungi are distributed between yeasts (6%) and moulds (3%) (Figure 2). Concerning the distribution of these germs according to the studied departments, a great germs diversification was observed according to each department. From the 27 isolated germs, 18 germs were isolated in the female surgery department, 13 in male surgery, 10 in gynecological-obstetrics, 9 in children's surgery, and 4 in the operating theater (Figure 3).

The distribution of isolated germs, according to the five studied departments, has shown their predominance in male and female surgery with a rate of 17%, followed by gynecological-obstetrics 11%, operating theater 9%, and then children's surgery 8%. Moreover, the *Staphylococcus* and *Streptococcus* species were the most frequent in all surgery departments (Figure 3).

### 3.2. Model Approach for Extracting Association Rules

Based on the application of the “algorithm a priori” on our transactional database (with min support 25%, min confidence 75%, and lift greater than 1), the obtained results show a strong association of contamination between the following:

- Wall and nightstand and door cuff at 100% confidence or with 100% confidence
- Ground and door cuff and bed rails with a confidence rate of 87%
- Door cuff and nightstand and bed rails on the one hand and the ground on the other hand in the confidence 77.7%

The quality of the lift indicator is strictly higher than 1, which means the importance and quality of the association rule used (Table 1).

Concerning the isolated germs, strong associations of microorganism's presence have been found between *Pseudomonas luteola* and coagulase-negative *Staphylococci* within a confidence interval of 100%, between *Staphylococcus aureus* and *Streptococcus* spp. and coagulase-negative *Staphylococci* with 90% confidence, and between *Escherichia coli* and *Staphylococcus aureus* on the one hand and coagulase-negative *Staphylococci* on the other hand, with respective confidence of 85% and 71%. Furthermore, the quality of the lift indicator of our model is also strictly superior to 1, which confirms the importance and quality of the association rule used (Table 2).

### 3.3. Evaluation of the Disinfection Procedure

Before disinfection, multiple and diversified presences of microorganisms have been found. Then, just after disinfection, a total absence of germs following the use of the 3 selected disinfectants has been shown. Two hours after disinfection, the presence of *Staphylococcus aureus* has been observed in ground surface 1 (disinfected with disinfectant 1), and *Streptococcus* bacterial strains have been found in ground surfaces 2 and 3 (disinfected with disinfectants 2 and 3).

But, four hours after disinfection, the samples have shown the presence and diversification of bacteria in the 3 sampling sites, and after 6 hours multiplication and diversification of bacteria and the presence of fungi (yeasts) have been noticed (Table 3).

- Disinfectant 1 based on quaternary ammonium: didecyl methyl polyoxyethyl ammonium propionate as active substance.
- Disinfectant 2 based on didecyl dimethyl ammonium chloride (CAS N: 7173-51-5): 3.5% w/w N-(3-aminopropyl)-N-dodecylpropane-1, 3-diamine (CAS N: 2372-82-9): 5.5% w/w, nonionic surfactants, EDTA and salts, allergen-free fragrance labeling.
- Disinfectant 3 based on nonionic surfactant, didecyl dimethyl ammonium chloride CAS no. 7173-51-5 (5% m/m), polyaminopropyl biguanide CAS no. 27083-27- 8 (1.6% m/m), and complexing agent.

## 4. Discussion

The hospital environment plays a very important role in the colonization and transmission of opportunistic pathogens. Moreover, the microorganisms responsible for hospital-acquired infections can survive for days, weeks, or even months on hospital surfaces [21, 22]. For this reason, it is necessary to carry out microbiological monitoring of the hospital environment to find out the strains circulating in this environment. Our study, which is to the best of our knowledge the first to be carried out in this hospital center, shows that 83% of sites are contaminated compared to 17% which are not. Moreover, results show the increase in surface contamination in the men's and women's surgery departments that could be justified by the free access of patients. The contamination of operating theater is justified by the poor quality of disinfection and cleaning procedures between surgical operations, despite the limited access of patients and nursing staff, and by the multitude of surgical specialties in these departments.

The most contaminated sites are the floor, door handles, and bed rails, due to their frequent use by patients and nursing staff, as these sites are close to the patient, and this has been justified by several studies [23, 24].

The majority of bacteria isolated from the different studied services' surfaces are Gram-positive germs. This result is similar to those reported in a study carried out in a hospital center in Fez and contradicts other studies [4, 13, 25]. This contradiction could be due to the hospital size (especially its bed capacity) and the studied department specificity, as our study was carried out more precisely in the surgery departments of a small hospital center, while the other studies were carried out in other departments. It could also be due to the nature of care lavished in the departments and the disinfection method, as well as the visitors' access to the departments especially the open access ones. According to Shmitt et al., the environment constitutes a reservoir for Gram-positive bacteria, widely dispersed by human activity. These bacteria are more resistant to desiccation than Gram-negative bacteria, especially if the surfaces undergo only twice daily cleaning [26].

This study has shown that the most existing strains belong to the Staphylococcus family (40%) and are distributed between *Staphylococcus aureus* (18%) and coagulase-negative *Staphylococcus* (22%). The presence of these two species could indicate a lack of hygiene in the hospital environment. The Staphylococci survive for several days on the surfaces [27]. Moreover, *Staphylococcus* has been found in a variety of sites including both community and hospital settings, particularly in surgery which can represent a very high risk of contracting an operative site infection for patients [28].

The second part of this work was based on the extraction model of the association rules, which has been applied for the first time, to the best of our knowledge, to surface sites and isolated germs in the hospital environment. The obtained results show that there are strong links between the contamination of the sampling sites and the isolated germs. This has made it possible to optimize the sampling, cleaning, and disinfection scenarios of the most contaminated sites, as well as knowing the behavior and profile of germs circulating in the different sites.

In the present study, a strong association was found between wall and nightstand's and door cuff's contaminations, meaning that the existence of contamination in wall and nightstand systematically indicates that door cuff is contaminated. Strong associations between ground and door cuff and bed rails were also found meaning that the existence of the contamination of ground and door cuff systematically indicates that bed rails are contaminated. Another strong association of contamination between door cuff and nightstand and bed rails means that the existence of contamination in door cuff and nightstand indicates systematically that bed rails are contaminated, and finally a strong association between door cuff and nightstand and ground means that the existence of door cuff and nightstand contamination systematically indicates that ground is contaminated. This increases the rate of optimization of intervention time and the effectiveness of disinfectant chosen (Table 1).

Concerning the germs' association, strong association of presence found between *Pseudomonas luteola* and coagulase-negative *Staphylococcus* means that the presence of *Pseudomonas luteola* indicates that coagulase-negative *Staphylococcus* exists. Moreover, the strong association between *Staphylococcus aureus*, *Streptococcus* spp., and coagulase-negative *Staphylococcus* shows that the presence of *Staphylococcus aureus* and *Streptococcus* spp. indicates the presence of coagulase-negative *Staphylococcus*.

There is a strong association between *Escherichia coli* or *Staphylococcus aureus* with coagulase-negative *Staphylococci* meaning that the presence of *Escherichia coli* as well as *Staphylococcus aureus* indicates the presence of coagulase-negative *Staphylococci*, which explains the nature and the profiles of germs circulating in the services. This association justifies the coexistence of bacteria within the same community and explains why each bacterial cell maintains a network of relations with its neighbors. Indeed, the cells of different bacterial species are not distributed homogeneously; they are rather modeled by their interactions with the neighboring cells and the abiotic environmental factors according to their metabolic and physiological needs [29].

The disinfection procedure evaluation after the use of three disinfectants has shown a total absence of germs immediately after the disinfection; the presence of Gram-positive bacteria 2 hours after disinfection; a multiple presence of Gram-positive and negative bacteria 4 h after, and the multiplication of bacteria and the presence of yeasts 6 hours after disinfection. These results suggest the necessity to renew the disinfection of each site every 4 hours to reduce the risk of hospital surfaces contamination and thereby the risk of nosocomial infections occurrence. These findings are consistent with several studies having shown that the regular disinfection of hospital environment reduces significantly the potential risk of nosocomial infection [30, 31], which demonstrates the role of the hospital environment in nosocomial infections and constitutes data support that can be used to identify the transmission mode of environmental microbes to patients.

## 5. Conclusion

This study allowed us to know the germs circulating in the different surgical departments, whose identification revealed the predominance of *Staphylococci* which can be caused by infection of the operating site. Moreover, strong associations of contamination of the sampling sites and the germs have been found indicating a cross-contamination from one site to another with diversification of germs, thanks to the application of an innovative model approach for extracting association rules. Concerning the evaluation of the disinfection procedure efficiency of the most contaminated points in hospital surfaces, results showed that it is necessary to repeat the disinfection procedure at least every 4 hours to control the risk of surface infection, and thereby the risk of nosocomial infections occurs.

## Figures and Tables

**Table 1:** The extraction model of the association rule between sampling points.

Antecedents	Consequents	Antecedent support	Consequent support	Support	Confidence	Lift	Leverage	Conviction
Door cuff	Nightstand	0.407407	0.555556	0.333333	0.818182	1.472727	0.106996	2.444444
Door cuff	Wall	0.407407	0.444444	0.296296	0.727273	1.636364	0.115226	2.037037
Door cuff	Ground	0.407407	0.666667	0.296296	0.727273	1.090909	0.024691	1.222222
Door cuff	Bed rails	0.407407	0.555556	0.333333	0.818182	1.472727	0.106996	2.444444
Wall, door cuff	Nightstand	0.296296	0.555556	0.259259	0.875000	1.575000	0.094650	3.555556
Wall, nightstand	Door cuff	0.259259	0.407407	0.259259	1.000000	2.454545	0.153635	inf
Door cuff, nightstand	Wall	0.333333	0.444444	0.259259	0.777778	1.750000	0.111111	2.500000
Door cuff, night stand	Bed rails	0.333333	0.555556	0.259259	0.777778	1.400000	0.074074	2.000000
Door cuff, bed rails	Nightstand	0.333333	0.555556	0.259259	0.777778	1.400000	0.074074	2.000000
Night stand, bed rails	Door cuff	0.333333	0.407407	0.259259	0.777778	1.909091	0.123457	2.666667
Ground, door cuff	Bed rails	0.296296	0.555556	0.259259	0.875000	1.575000	0.094650	3.555556
Ground, bed rails	Door cuff	0.333333	0.407407	0.259259	0.777778	1.909091	0.123457	2.666667
Door cuff, nightstand	Ground	0.333333	0.666667	0.259259	0.777778	1.166667	0.037037	1.500000

**Table 2:** The extraction model of the association rule between isolated germs.

Antecedents	Consequents	Antecedent support	Consequent support	Support	Confidence	Lift	Leverage	Conviction
*Staphylococcus aureus*	Coagulase-negative *Staphylococci*	0.58	0.70	0.50	0.862069	1.231527	0.0940	2.175
Coagulase-negative *Staphylococci*	*Staphylococcus aureus*	0.70	0.58	0.50	0.714286	1.231527	0.0940	1.470
*Streptococcus* spp.	*Staphylococcus aureus*	0.36	0.58	0.22	0.611111	1.053640	0.0112	1.080
*Escherichia coli*	*Staphylococcus aureus*	0.14	0.58	0.12	0.857143	1.477833	0.0388	2.940
*Pseudomonas luteola*	Coagulase-negative *Staphylococcus*	0.12	0.70	0.12	1.000000	1.428571	0.0360	inf
*Streptococcus* spp.	Coagulase-negative *Staphylococcis*	0.36	0.70	0.28	0.777778	1.111111	0.0280	1.350
*Escherichia coli*	Coagulase-negative *Staphylococci*	0.14	0.70	0.10	0.714286	1.020408	0.0020	1.050
*Staphylococcus aureus, Streptococcus* spp.	Coagulase-negative *Staphylococci*	0.22	0.70	0.20	0.909091	1.298701	0.0460	3.300
*Streptococcus* spp., Coagulase-negative *Staphylococci*	*Staphylococcus aureus*	0.28	0.58	0.20	0.714286	1.231527	0.0376	1.470

**Table 3:** Evaluation of the disinfection procedure according to time and contaminated points.

	Sampling points		
	Sampling point 1	Sampling point 1	Sampling point 1
Before using disinfectant	(i) *Staphylococcus aureus*	(i) *Staphylococcus aureus*	(i) Coagulase-negative *Staphylococci*
	(ii) *Streptococcus* spp.	(ii) *Streptococcus* spp.	(ii) *Streptococcus* spp.
	(iii) *Pantoea* spp.	(iii) *Diplocoques* Gram+	(iii) Yeasts
	(iv) Yeasts		

	Use of disinfectants		
	Disinfectant 1	Disinfectant 2	Disinfectant 3

After using disinfectant	Absence of germs	Absence of germs	Absence of germs
Two hours after using disinfectant	(i) *Staphylococcus aureus*	(i) *Staphylococcus aureus*	(i) Coagulase^*-*^*Staphylococci*
		(ii) *Streptococcus* spp.	(ii) *Staphylococcus aureus*

Four hours after using disinfectant	(i) *Staphylococcus aureus*	(i) *Streptococcus* spp.	(i) Coagulase-negative *Staphylococci Streptococcus* spp.
	(ii) *Streptococcus* spp.	(ii) *Enterococcus* spp.	(ii) *Enterococcus* spp.
		(iii) *Staphylococcus aureus*	

Six hours after using disinfectant	(i) *Staphylococcus aureus*	(i) *Staphylococcus aureus*	(i) Coagulase-negative *Staphylococci Streptococcus* spp.
	(ii) Coagulase-negative *Staphylococci*	(ii) *Streptococcus* spp.	(ii) *Enterococcus* spp.
	(iii) *Streptococcus* spp.	(iii) *Coagulase* - *Staphylococci*	(iii) Yeasts
	(iv) *Pantoea spp*	(iv) *Pseudomonas luteola*	
	(v) Yeasts	(v) Yeasts	

## Data Availability

The data used to support the findings of this study are available from the corresponding author upon request.
